# ULTRASOUND MUSCLE THICKNESS AS AN EASY METHOD TO ASSESS LEAN MASS AND GRIP STRENGTH IN OLDER ADULTS

**DOI:** 10.1093/geroni/igac059.2996

**Published:** 2022-12-20

**Authors:** Lara Vilar Fernandes, Gabriela Benatti De Oliveira, Ana Carolina Junqueira Vasques, Flavia Andrade, Xia Yu, Summer Chen, Ligiana P Corona

**Affiliations:** University of Campinas - UNICAMP, Limeira, Sao Paulo, Brazil; University of Campinas - UNICAMP, Limeira, Sao Paulo, Brazil; University of Campinas - UNICAMP, Limeira, Sao Paulo, Brazil; University of Illinois--Urbana-Champaign, Urbana, Illinois, United States; University of Campinas - UNICAMP, Limeira, Sao Paulo, Brazil; University of Illinois at Urbana Champaign, Urbana, Illinois, United States; Universidade Estadual de Campinas/ Faculdade de Ciências Aplicadas, Limeira, Sao Paulo, Brazil

## Abstract

Ultrasound is emerging as an effective method for measuring muscle mass in older adults, being more cost-effective than other image methods, such as Dual-energy X-ray absorptiometry (DXA) and computed tomography, and more accurate than bioelectrical impedance and anthropometry. We aimed to evaluate the correlation between measurements of muscle thickness (MT) measured by a portable A-mode ultrasound, the total lean mass (TLM) measured by DXA, and grip strength (GS) among community-dwelling Brazilian older adults. This is a cross-sectional pilot study with 30 participants (9 men and 21 women) aged 60–78. MT was assessed in five anatomic sites (triceps, biceps, anterior and posterior thigh, calf) using a portable A-mode ultrasound (BodyMetrix BX-2000 - IntelaMetrix, Inc.); TLM was assessed by DXA (GE Healthcare’s Lunar iDXA), and GS was assessed using a Saehan dynamometer. Pearson′s test was used for correlation analysis between each site MT and TLM; linear regressions were estimated to verify the association between GS and each site MT, controlling for age. Lower limb measurements (anterior and posterior thigh, calf) were not associated with TLM and GP. Triceps MT was significantly correlated to TLM (r=0.565; p=0.001) and associated to GS (β=0.699; p=0.004); biceps MT was also correlated to TLM (r=0.425; p=0.019) and associated to GS (β=0.652; p=0.012). Our results suggest that upper limb ultrasound measurements using portable equipment can be a helpful cost-effective indicator of muscle mass and function in older adults. Further results and conclusions will be reported after completing the study with the total planned sample (n=150).

